# Factors associated with knowledge about pharmacological management of pregnant women in Peruvian dental students: a logistic regression analysis

**DOI:** 10.1186/s12909-023-04068-9

**Published:** 2023-02-04

**Authors:** Elizabeth Flores-Montalvo, Nancy Córdova-Limaylla, Marysela Ladera-Castañeda, Carlos López-Gurreonero, Alí Echavarría-Gálvez, Alberto Cornejo-Pinto, Luis Cervantes-Ganoza, César Cayo-Rojas

**Affiliations:** 1 Universidad Privada San Juan Bautista, School of Stomatology, Av. Jose Antonio Lavalle Avenue s/n (Ex Hacienda Villa); Chorrillos, 15066 Lima, Peru; 2 Universidad Peruana de Ciencias Aplicadas, Academic Program of Dentistry, 15066 Lima, Peru; 3 Universidad Nacional Federico Villarreal, Faculty of Dentistry and Postgraduate School, Research team “Salud Pública - Salud Integral”, 15084 Lima, Peru; 4 Universidad Científica del Sur, School of Stomatology, 15067 Lima, Peru; 5 Faculty of Stomatology, Universidad Inca Garcilaso de la Vega, 15084 Lima, Peru

**Keywords:** Knowledge, Logit model, Pregnancy, Pharmacology, Dentistry, Peru

## Abstract

**Background:**

Clinical management to maintain or restore oral health through the use of drugs during pregnancy is crucial, since at this stage physiological changes significantly influence the absorption, distribution and elimination of the drug, considering also that excessive administration of drugs during this period may have adverse effects on the mother and/or fetus. Therefore, the aim of the present study was to evaluate the factors associated with knowledge of pharmacological management of pregnant women in dental students of a Peruvian university located in the capital and province.

**Methods:**

This analytical, cross-sectional, prospective and observational study assessed 312 Peruvian dental students from third to fifth year of study between February and April 2022. A validated questionnaire of 10 closed questions was used to measure knowledge about pharmacological management in pregnant women. A logit model was used to assess the influence of the variables: gender, age, year of study, marital status, place of origin and area of residence. A significance of *p* < 0.05 was considered.

**Results:**

The 25.96, 55.13 and 18.91% of the dental students showed poor, fair and good knowledge about pharmacological management in pregnant women; respectively. In addition, it was observed that students under 24 years of age and those from the capital were significantly (*p* < 0.05) 44% less likely to have poor knowledge of pharmacological management in pregnant women compared to those aged 24 years or older (OR = 0.56; CI: 0.34–0.92) and those from the province (OR = 0.56; CI: 0.32–0.98); respectively. Finally, those in their third and fourth year of study were significantly three times more likely to have poor knowledge (OR = 3.17; CI: 1.68–5.97 and OR = 3.88; CI: 2.07–7.31; respectively) compared to fifth year dental students.

**Conclusion:**

The knowledge of dental students about pharmacological management in pregnant women was predominantly of fair level. In addition, it was observed that being under 24 years of age and being from the capital city were protective factors against poor knowledge, while being a third- and fourth-year student was a risk factor. Finally, gender, marital status and area of residence were not influential factors in the level of knowledge.

## Background

Oral health during pregnancy is critical to ensure optimal fetal development, which in turn contributes to the overall wellbeing of pregnant women [1–5]. There is growing evidence that improper dental treatment and drug therapy are associated with adverse pregnancy outcomes, including infant malformations or miscarriages [6, 7]. During pregnancy, many changes occur in the oral cavity that may be related to periodontal diseases such as gingivitis and periodontitis, as there is a link between increased plasma levels of pregnancy hormones and decreased periodontal health [8]. Therefore, both dentists and pregnant women need to be aware of the repercussions that these physiological changes may have on the oral cavity.

Recent studies show that due to lack of knowledge and information on oral health provided by dentists, about 50% of women do not go to the dentist during pregnancy [7, 9, 10]. Likewise, another report indicates that dentists have been reluctant to treat pregnant patients due to uncertainty about the risks to the mother and fetus [6]. Given the substantial importance of this issue, it is worth investigating how much information dental students receive during their professional training in order to provide appropriate pharmacological prescribing with an understanding of the metabolic and physiological changes that occur during pregnancy [11, 12].

Around 90% of women take at least some medication and 50% take at least 4 medications during pregnancy [13]. Currently, 5% of pregnant women suffer from certain chronic diseases such as asthma, chronic arterial hypertension, diabetes, among others, and must follow some pharmacological treatment [9]. The Food and Drug Administration (FDA) has assigned risk categories for drugs during pregnancy (A, B, C, D and X). Regarding category A and B drugs, evidence has shown that they can be safe in pregnant women. However, category C and D drugs should only be prescribed in strictly necessary cases. Finally, drugs in category X should not be prescribed under any circumstances in pregnant women [14–16].

On the other hand, some authors have reported that sociodemographic factors such as age, sex and year of study have been associated with the level of knowledge presented by some students regarding pharmacological management in pregnant women [11, 17].

To date (August 2022), very few studies have been conducted to assess the association between sociodemographic factors and dentists’ level of knowledge about prescribing drugs during pregnancy. For example, Guevara et al reported that the level of dental students was fair [11]; while Razban et al reported that Swiss dentists had good knowledge for providing dental care to pregnant women [18].

Therefore, the aim of the present study was to assess the sociodemographic factors associated with knowledge about pharmacological management of pregnant women in dental students at a Peruvian university.

## Methods

### Type of study and delimitation

This analytical, observational, cross-sectional and prospective study was conducted from February to April 2022 at the School of Dentistry of the Universidad Privada San Juan Bautista (UPSJB), based in the Peruvian capital (Lima) and a branch in the province (Ica). This manuscript was written according to the STrengthening the Reporting of OBservational studies in Epidemiology (STROBE) guidelines for observational studies [19].

### Population and selection of participants

The total population consisted of dental students from a private university located in the Peruvian capital (Lima) with a branch in the province of Ica, Peru. These students were in their third, fourth and fifth year of their professional careers and carried out their theoretical classes completely virtually due to the context of the COVID-19 pandemic, while their pre-professional practices were carried out in person, both in laboratories, teaching clinics and hospitals in the case of the fifth year students who were doing their internships. The 1st and 2nd year students were not included, since according to their curricular plan they do not take subjects that include pharmacology-related topics, as they mostly take basic training subjects.

The aforementioned population consisted of 322 UPSJB Dentistry students, with 121 students in the 3rd year of study, 111 students in the 4th year of study and 90 students in the 5th year of study. Finally, no sample size calculation was required since the entire target population of 312 students (117 [3rd year students], 108 [4th year students] and 87 [5th year students]) was included in the study according to the following eligibility criteria:

#### Inclusion criteria

- Students from the Academic Program of Dentistry of the UPSJB who are enrolled from third to fifth year of study in the 2022-I semester.
- Students who gave informed and voluntary consent to participate in the study.

#### Exclusion criteria

- Students who voluntarily withdrew while the study was in progress.

### Variables

In the present study, the dependent variable was knowledge about pharmacological management in pregnant women and the independent variables were sex, age and year of study [11, 17], with marital status, place of origin and area of residence as possible confounding variables.

### Preparation of the instrument

A questionnaire of 10 closed questions with polytomous answers (Yes / No / Don’t know) was prepared to assess the knowledge about the pharmacological management of pregnant women in dental students, with 10 questions (Q1 to Q10). The level of knowledge was defined according to the following scale: poor (0–3 points), fair (4–6 points) and good (7–10 points). One point was awarded for each correct answer. To estimate the level of knowledge (poor, fair and good), the Stanones scale [mean total score ± 0.75 (standard deviation)] was used to establish cut-off points [Table 1].

**Table 1:** Questionnaire

Questions	
**Q1.** Is dental treatment recommended for pregnant women during the second trimester?	
**Q2.** Are NSAIDs safe to use during pregnancy?	
**Q3.** According to the FDA classification of risk in pregnancy, does tetracycline cause miscarriage and congenital heart disease?	
**Q4.** Are cephalosporins contraindicated antibiotics in pregnant women?	
**Q5**. Is gingivitis the most frequent oral pathology in pregnancy?	
**Q6.** Is acetylsalicylic acid a recommended analgesic for dental use during pregnancy?	
**Q7.** Can amoxicillin be indicated in pregnant women?	
**Q8.** In pregnant women allergic to penicillin, can Clindamycin be an antibiotic alternative in the dental practice?	
**Q9.** Is miconazole 2% cream an antifungal of choice to treat oral candidiasis in pregnant women?	
**Q10.** Can lidocaine be used as local anaesthesia for dental treatment in the second trimester of pregnancy?	

*FDA*, Food and Drug Administration, *NSAIDs *Non-steroidal anti-inflammatory drugs

### Validation of the instrument

The content validity of the questionnaire was acceptable with Aiken’s V (0.87; CI: 0.82–0.90) as judged by three experts with more than 10 years of experience in research, oral surgery and pharmacology, who assessed the clarity, objectivity, timeliness, organization, sufficiency, intentionality, consistency, coherence and methodology of the instrument. For construct validity, a factor analysis was performed with answers from 100 randomly selected participants (*n* = 10 k, [10 participants minimum per item]) [20] to define the dimensions and group the items, establishing a single dimension (Q1 to Q10). Subsequently, the internal consistency reliability of the instrument was assessed by means of the Kuder-Richardson test (KR-20), obtaining a result of 0.73, which proved to be acceptable. In addition, a group of 30 randomly selected students were given the questionnaire at two different times within 10 days to assess the reproducibility of the instrument altering the order of the questions to avoid recall bias (test-retest) [21], being the intraclass correlation coefficient very good (ICC = 0.88; 95% CI: 0.76–0.94).

### Procedure

The questionnaire was distributed to each student via e-mail using the educational web service *Google Classroom*®. The invitation to participate was made by the principal investigator (E.F.M) providing her full name, university and contact details such as institutional email and telephone. In some cases, it was necessary to resend the invitation once a week up to a maximum of three times. The informed consent to participate in the study was placed at the beginning of the instrument followed by the indications to develop the questionnaire. However, students were free to refuse the assessment if they did not wish to complete it during its course. The principal researcher had access to personal data such as telephone number and name. Only one submission per student was considered. In addition, no incentives were offered for participation in this study.

### Statistical analysis

The data analysis was carried out with completed surveys, using the Statistical Package for the Social Sciences (SPSS) version 28.0. Descriptive statistics were applied to use frequency table and bar graphs. Pearson’s chi-square test was used for bivariate analysis, and for expected values less than 5, Fisher’s exact test was used. Influencing factors were established with the logistic regression model (logit model) using odds ratio (OR). All analyses were performed, considering a significance level of 5% (*p* < 0.05).

### Bioethical considerations

The present research respected the bioethical principles of the Declaration of Helsinki related to confidentiality, freedom, respect, and nonmaleficence. In addition, we had the approval of an institutional ethics committee from the Universidad Privada San Juan Bautista with resolution No. 1524–2021-CIEI-UPSJB. Finally, an informed and voluntary consent was requested on the first page of the virtual questionnaire.

## Results

The response rate to the survey was 96.89% and the mean age of the 312 dental students was 25.5 ± 4.5 years, with a median age of 24 years. The female sex was the most frequent with 63.8% of the total number of participants. The predominant age group was under 24 years of age (55.8%). Most of dental students were in their third year (37.5%). The highest percentage of participants were unmarried (88.5%). In addition, 70.8% were from the capital city and 93.3% lived in urban areas [Table 2].

**Table 2:** Sociodemographic characteristics of dental students from a Peruvian university

Variable	Category	Frequency	Percentage
**Sex**	Female	199	63.8
	Male	113	36.2
**Age group**	< 24 years	174	55.8
	≥ 24 years	138	44.2
**Year of study**	3rd year	117	37.5
	4th year	108	34.6
	5th year	87	27.9
**Marital status**	Unmarried	276	88.5
	Married	36	11.5
**Place of origin**	Capital city	221	70.8
	Province	91	29.2
**Area of residence**	Urban	291	93.3
	Rural	21	6.7

*SD* Standard Deviation

The majority of correct answers between males and females differed by less than 5.0%, with the exception of Q3 and Q4 where females had a higher percentage of correct answers at 10.0 and 7.4%, respectively. The percentage differences in correct answers between those aged 24 and under and those aged 24 years or older did not exceed 10.0%, with the exception of Q1 and Q3, as those aged 24 and under had a higher percentage of correct answers at 10.9% for Q1, while those aged 24 years or older had a higher percentage of correct answers for Q3 at 11.5%. On the other hand, the highest percentage of correct answers in 7 of the 10 questions was obtained by 5th year students, with a percentage of over 52.0%. The percentage difference of correct answers between unmarried and married students was higher than 10% for Q2, Q9 and Q10 in favour of married students and only for Q2 in favour of unmarried students. Likewise, the percentage difference between students from the capital and the province was greater than 10% for Q1, Q5 and Q8, in favour of those from the capital. Finally, it could be observed that the percentage differences between those residing in urban and rural areas were greater than 10% for Q4, in favour of the urban area; and for Q2 and Q10, in favour of the rural area [Table 3].

**Table 3:** Knowledge of pharmacological management in pregnant women among dental students at a Peruvian university

Variable	Category	Q1		Q2		Q3		Q4		Q5		Q6		Q7		Q8		Q9		Q10	
		I	C	I	C	I	C	I	C	I	C	I	C	I	C	I	C	I	C	I	C
		f (%)	f (%)	f (%)	f (%)	f (%)	f (%)	f (%)	f (%)	f (%)	f (%)	f (%)	f (%)	f (%)	f (%)	f (%)	f (%)	f (%)	f (%)	f (%)	f (%)
**Sex**	Female	85 (42.7)	114 (57.3)	90 (45.2)	109 (54.8)	121 (60.8)	78 (39.2)	112 (56.3)	87 (43.7)	80 (40.2)	119 (59.8)	92 (46.2)	107 (53.8)	109 (54.8)	90 (45.2)	109 (54.8)	90 (45.2)	117 (58.8)	82 (41.2)	96 (48.2)	103 (51.8)
	Male	43 (38.1)	70 (61.9)	48 (42.5)	65 (57.5)	80 (70.8)	33 (29.2)	72 (63.7)	41 (36.3)	44 (38.9)	69 (61.1)	65 (57.5)	48 (42.5)	64 (56.6)	49 (43.4)	60 (53.1)	53 (46.9)	64 (56.6)	49 (43.4)	55 (48.7)	58 (51.3)
	*p	0.421		0.639		0.076		0.199		0.827		0.055		0.75		0.775		0.711		0.942	
**Age group**	< 24 years	63 (36.2)	111 (63.8)	71 (40.8)	103 (59.2)	121 (69.5)	53 (30.5)	99 (56.9)	75 (43.1)	71 (40.8)	103 (59.2)	94 (54.0)	80 (46.0)	95 (54.6)	79 (45.4)	91 (52.3)	83 (47.7)	99 (56.9)	75 (43.1)	89 (51.1)	85 (48.9)
	≥ 24 years	65 (47.1)	73 (52.9)	67 (48.6)	71 (51.4)	80 (58.0)	58 (42.0)	85 (61.6)	53 (38.4)	53 (38.4)	85 (61.6)	63 (45.7)	75 (54.3)	78 (56.5)	60 (43.5)	78 (56.5)	60 (43.5)	82 (59.4)	56 (40.6)	62 (44.9)	76 (55.1)
	*p	0.052		0.171		0.034*		0.402		0.667		0.142		0.734		0.457		0.654		0.275	
**Year of study**	3rd year	41 (35.0)	76 (65.0)	50 (42.7)	67 (57.3)	69 (59.0)	48 (41.0)	67 (57.3)	50 (42.7)	52 (44.4)	65 (55.6)	64 (54.7)	53 (45.3)	76 (65.0)	41 (35.0)	62 (53.0)	55 (47.0)	83 (70.9)	34 (29.1)	55 (47.0)	62 (53.0)
	4th year	54 (50.0)	54 (50.0)	51 (47.2)	57 (52.8)	64 (59.3)	44 (40.7)	65 (60.2)	43 (39.8)	53 (49.1)	55 (50.9)	52 (48.1)	56 (51.9)	66 (61.1)	42 (38.9)	72 (66.7)	36 (33.3)	59 (54.6)	49 (45.4)	64 (59.3)	44 (40.7)
	5th year	33 (37.9)	54 (62.1)	37 (42.5)	50 (57.5)	68 (78.2)	19 (21.8)	52 (59.8)	35 (40.2)	19 (21.8)	68 (78.2)	41 (47.1)	46 (52.9)	31 (35.6)	56 (64.4)	35 (40.2)	52 (59.8)	39 (44.8)	48 (55.2)	32 (36.8)	55 (63.2)
	*p	0.059		0.741		0.007*		0.892		< 0.001*		0.483		< 0.001*		0.001*		0.001*		0.007*	
**Marital status**	Unmarried	111 (40.2)	165 (59.8)	126 (45.7)	150 (54.3)	173 (62.7)	103 (37.3)	163 (59.1)	113 (40.9)	112 (40.6)	164 (59.4)	141 (51.1)	135 (48.9)	153 (55.4)	123 (44.6)	152 (55.1)	124 (44.9)	164 (59.4)	112 (40.6)	143 (51.8)	133 (48.2)
	Married	17 (47.2)	19 (52.8)	12 (33.3)	24 (66.7)	28 (77.8)	8 (22.2)	21 (58.3)	15 (41.7)	12 (33.3)	24 (66.7)	16 (44.4)	20 (55.6)	20 (55.6)	16 (44.4)	17 (47.2)	19 (52.8)	17 (47.2)	19 (52.8)	8 (22.2)	28 (77.8)
	*p	0.422		0.162		0.075		0.934		0.403		0.453		0.989		0.374		0.163		0.001*	
**Place of origin**	Capital	79 (35.7)	142 (64.3)	98 (44.3)	123 (55.7)	140 (63.3)	81 (36.7)	129 (58.4)	92 (41.6)	81 (36.7)	140 (63.3)	110 (49.8)	111 (50.2)	124 (56.1)	97 (43.9)	112 (50.7)	109 (49.3)	123 (55.7)	98 (44.3)	106 (48.0)	115 (52.0)
	Province	49 (53.8)	42 (46.2)	40 (44.0)	51 (56.0)	61 (67.0)	30 (33.0)	55 (60.4)	36 (39.6)	43 (47.3)	48 (52.7)	47 (51.6)	44 (48.4)	49 (53.8)	42 (46.2)	57 (62.6)	34 (37.4)	58 (63.7)	33 (36.3)	45 (49.5)	46 (50.5)
	*p	0.003*		0.950		0.537		0.736		0.082		0.763		0.715		0.054		0.189		0.811	
**Area of residence**	Urban	118 (40.5)	173 (59.5)	131 (45.0)	160 (55.0)	186 (63.9)	105 (36.1)	169 (58.1)	122 (41.9)	117 (40.2)	174 (59.8)	146 (50.2)	145 (49.8)	163 (56.0)	128 (44.0)	158 (54.3)	133 (45.7)	167 (57.4)	124 (42.6)	143 (49.1)	148 (50.9)
	Rural	10 (47.6)	11 (52.4)	7 (33.3)	14 (66.7)	15 (71.4)	6 (28.6)	15 (71.4)	6 (28.6)	7 (33.3)	14 (66.7)	11 (52.4)	10 (47.6)	10 (47.6)	11 (52.4)	11 (52.4)	10 (47.6)	14 (66.7)	7 (33.3)	8 (38.1)	13 (61.9)
	*p	0.525		0.298		0.487		0.230		0.534		0.845		0.455		0.865		0.405		0.328	
**Total**		128 (41.0)	184 (59.0)	138 (44.2)	174 (55.8)	201 (64.4)	111 (35.6)	184 (59.0)	128 (41.0)	124 (39.7)	188 (60.3)	157 (50.3)	155 (49.7)	173 (55.4)	139 (44.6)	169 (54.2)	143 (45.8)	181 (58.0)	131 (42.0)	151 (48.4)	161 (51.6)

*I* Incorrect response,  *C* Correct response; f: absolute frequency, and (%): relative frequency; *p < 0.05 (significant association based on Pearson’s Chi-square)

Regarding knowledge about pharmacological management in pregnant women among dental students, statistically significant associations were obtained for the age group with Q3 (*p* = 0.034). Year of study was significantly associated with Q3, Q5, Q7, Q8, Q9 and Q10 (*p* = 0.007, *p* < 0.001, *p* < 0.001, *p* = 0.001, *p* = 0.001 and p = 0.007, respectively). In addition, marital status was significantly associated with Q10 (*p* = 0.001). Finally, place of origin was significantly associated with Q1 (*p* = 0.003) [Table 3].

**Fig. 1:**
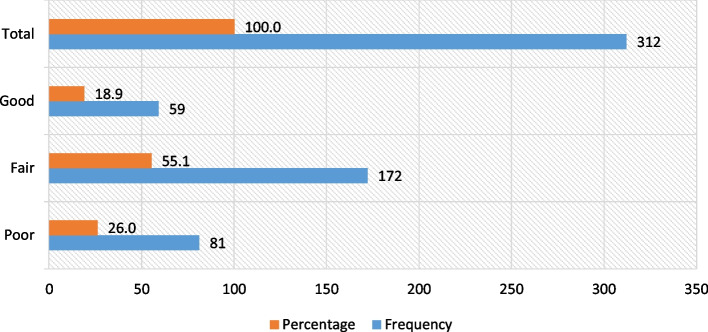
Frequency of the level of knowledge about pharmacological management of pregnant women among dental students at a Peruvian university

Of the 312 dental students surveyed, 25.96% showed poor knowledge, while 55.13% showed fair knowledge and 18.91% showed good knowledge about pharmacological management of pregnant women **[**Fig. 1**].**

The differences in the relative frequency of knowledge level (poor, fair and good) between females and males were 3.7, 0.4 and 3.3%, respectively. The differences between students under 24 and aged 24 years or older were 2.4, 3.8 and 1.4%, respectively. The differences between unmarried and married students were 7.4, 2.6 and 10.0%, respectively. Similarly, among those from the capital or the province, the differences were 13.0, 12.6 and 0.3%, respectively. For those living in urban or rural areas, the differences were 2.3, 13.1 and 15.4%, respectively. Finally, the highest frequencies of poor, fair and good levels of knowledge were found among students in the 4th year (32.4%), 3rd year (62.4%) and 5th year (34.5%) of the professional career, respectively. On the other hand, it was observed that the level of knowledge about pharmacological management in pregnant women was significantly associated with year of study (*p* < 0.001) and place of origin (*p* = 0.048) of dental students (*p* = 0.048) [Table 4].

**Table 4:** Association of sociodemographic factors of dental students with the level of knowledge about pharmacological management in pregnant women

Variable	Category	Level of knowledge			*p
		Poor	Fair	Good	
		f (%)	f (%)	f (%)	
**Sex**	Female	49 (24.6)	110 (55.3)	40 (20.1)	0.673
	Male	32 (28.3)	62 (54.9)	19 (16.8)	
**Age group**	< 24 years	47 (27.0)	93 (53.4)	34 (19.5)	0.798
	≥ 24 years	34 (24.6)	79 (57.2)	25 (18.1)	
**Year of study**	3rd year	29 (24.8)	73 (62.4)	15 (12.8)	< 0.001*
	4th year	35 (32.4)	59 (54.6)	14 (13.0)	
	5th year	17 (19.5)	40 (46.0)	30 (34.5)	
**Marital status**	Unmarried	74 (26.8)	153 (55.4)	49 (17.8)	0.301
	Married	7 (19.4)	19 (52.8)	10 (27.8)	
**Place of origin**	Capital	49 (22.2)	130 (58.8)	42 (19.0)	0.048*
	Province	32 (35.2)	42 (46.2)	17 (18.7)	
**Area of residence**	Urban	76 (26.1)	163 (56.0)	52 (17.9)	0.209
	Rural	5 (23.8)	9 (42.9)	7 (33.3)	

f: absolute frequency, and (%): relative frequency. * Based on Pearson’s Chi-square, *p* < 0.05 (significant association)

According to the crude logistic regression model, knowledge about pharmacological management in pregnant women was considered as a dependent variable (dichotomised as poor = 1 and fair/good = 0); sex and age as independent variables; and year of study, marital status, place of origin and area of residence as possible confounding variables. As a result, age (*p* = 0.016), year of study (*p* < 0.001) and place of origin (*p* = 0.042) were obtained as significant influencing factors. Subsequently, the adjusted model showed that dental students under 24 years of age and those from the capital city were significantly 44% less likely to have poor knowledge about pharmacological management of pregnant women than those aged 24 years or older (OR = 0.56; CI: 0.34–0.92) (*p* = 0.023) and those from the provinces (OR = 0.56, CI: 0.32–0.98) (*p* = 0.042). Finally, dental students who were in their 3rd and 4th year of study were significantly (*p* < 0.001) three times more likely to have poor knowledge (OR = 3.17, CI: 1.68–5.97 and OR = 3.88, CI: 2.07–7.31; respectively), compared to those in their 5th year of study [Table 5].

**Table 5:** Logistic regression model of knowledge about pharmacological management in pregnant women according to associated factors

Variable	Category	Crude model					Adjusted model^a^			
		OR	95% CI		***p***	β	OR	95% CI		***p***
			LL	UL				LL	UL	
**Sex**	Female	0.91	0.55	1.51	0.710					
	Male	*Ref.*								
**Age**	< 24 years	0.52	0.30	0.89	0.016	- 0.581	0.56	0.34	0.92	0.023
	≥ 24 years	*Ref.*					*Ref.*			
**Year of study**	3rd year	3.28	1.71	6.30	< 0.001	1.154	3.17	1.68	5.97	< 0.001
	4th year	3.78	1.99	7.18	< 0.001	1.357	3.88	2.07	7.31	< 0.001
	5th year	*Ref.*					*Ref.*			
**Marital status**	Unmarried	1.39	0.63	3.06	0.414					
	Married	*Ref.*								
**Place of origin**	Capital	0.49	0.27	0.91	0.024	- 0.586	0.56	0.32	0.98	0.042
	Province	*Ref.*					*Ref.*			
**Area of residence**	Urban	1.82	0.63	5.26	0.269					
	Rural	*Ref.*								
Constant						- 3.017				0.001

^a^Adjusted logit model for all variables that resulted with *p* < 0.05 value in the crude model; *OR* Odds ratio, *95% CI* 95% confidence interval. For the adjusted model on knowledge about pharmacological management of pregnant women in dental students, *p* < 0.001 (significant for the omnibus test of the model coefficient); β: coefficient of determination

## Discussion

Oral health in pregnant women is altered by hormonal changes that result in increased permeability of the oral blood vessels and decreased immunity, making them more vulnerable to infections [22]. For treatment of these oral pathologies it is often necessary to prescribe medications, which could put the general health of pregnant women and their fetus at risk [23], so it is important to have adequate knowledge about the benefits and risks of each drug in order to avoid unwanted complications. Therefore, the aim of the present study was to determine the association of sociodemographic factors with the level of knowledge about the pharmacological management of pregnant women in dental students from a Peruvian university.

In the present study, it was found that the level of knowledge about the pharmacological management of pregnant women in dental students from a Peruvian university was predominantly fair with 55.13% of the total, which agrees with what was reported by Guevara et al [11], since they found that the level of knowledge of preclinical and clinical students about dental management of pregnant patients was mostly fair. In addition, in the present study, under the logit model, it was found that being younger than 24 years of age was a protective factor against poor knowledge in dental students. This can be explained by the fact that students over 24 years of age generally have more family, economic and work responsibilities, which may take time away from their academic preparation. Some of these students are married or cohabitating and even have children, unlike younger students where most of them have enough time for their educational preparation since in most cases their parents cover their basic needs [24].

In the present study, it was found that students from the capital city had a higher level of knowledge than those from the provinces. This could be explained by the fact that students from the capital city have the possibility of doing more rotations in public or private hospitals. Universities located in the capital normally have access to a large number of agreements with health institutions, which allow students to broaden their educational horizons, while in the Peruvian provinces the number of hospitals is quite limited. On the other hand, being in the 3rd and 4th years of academic training was a risk factor for having poor knowledge about dental management in pregnant women, compared to those who were in the 5th year. This could be based on the fact that learning is developed through a set of theoretical and practical activities during a formative process. Therefore, over time the student is able to increase the development of his or her skills and mastery of competencies that will allow him or her to have a high probability of passing general pharmacology exams compared to previous years [25]. This is in agreement with the study conducted by Alhemrani et al in which they reported that students who were still taking clinical and preclinical courses at university showed a fair level of knowledge about a dental area, while the majority of 5th year students showed a good level on the same topic [26].

In the present study, it was observed that there was an association between place of origin, year of study and age of dental students with their level of knowledge about pharmacological management in pregnant women. This is consistent with the results obtained by Taybeh et al, who reported that students in their last year had greater knowledge about the use of medications in pregnancy than those in previous years [17].

Likewise, in the present study, gender was not considered an influential factor in the level of knowledge about pharmacological management in pregnant women. This could be due to the fact that, at the time of the questionnaire, the students were in a virtual learning environment, which in some cases has been shown to improve student learning, regardless of gender, facilitating the acquisition of knowledge and decision-making [27].

The results obtained in the present study should be taken into consideration by professors of the different subjects in the dental fields [11, 12], specially by those who are linked to pharmacology, since it represents one of the most important areas of knowledge for students. In his or her professional life, the student will have a direct, legal and ethical responsibility to know the adverse effects, interactions, indications and contraindications of the different groups of drugs, especially in vulnerable patients such as pregnant women, in whom pharmacological action does not operate in the same way as in normal conditions due to the physiological changes that they undergo [14, 15]. Therefore, it is of utmost importance that dental students learn to correctly prescribe drugs, taking into account the trimester of gestation in which there are risks, in order to avoid teratogenic alterations that affect the health of the mother or fetus [6, 28, 29]. According to the Food and Drug Administration (FDA), type C drugs, although they do not increase the spontaneous incidence of birth defects, could potentially alter the normal course of pregnancy and/or injure the fetus or newborn. In addition, the FDA warns that type D drugs, such as tetracyclines, may cause maternal and/or fetal hepatoxicity, as well as damage to dental enamel and fetal bone growth. Finally, the FDA does not recommend type X drugs during pregnancy because it has categorically demonstrated that the harm caused by their use far exceeds the benefits [28–30].

The dose of the drug, the route of administration, the duration of treatment and the time of gestation are decisive in preventing teratogenic risks [31]. This should be taken into account by the dental student when prescribing nonsteroidal anti-inflammatory drugs (NSAIDs) such as Ibuprofen and dexketoprofen, since they are potent inhibitors of the synthesis of prostaglandins responsible for maintaining the patency of the fetal ductus arteriosus. Their consumption in the last months of pregnancy may lead to an increased risk of congenital anomalies in the fetus, especially in the circulatory system [28–30].

On the other hand, 48.4% of the total surveyed answered incorrectly about the use of lidocaine in pregnant women according to the trimester of pregnancy, which is worrisome since the student should know that according to the FDA, local anesthetics such as lidocaine and prilocaine have not demonstrated teratogenic effects in human and animal studies, unlike bupivacaine, articaine and mepivacaine that have shown some teratogenic risk.

In addition, it is important for the student to know that in order to perform dental procedures involving the use of an anesthetic, the first trimester of pregnancy represents a greater threat of teratogenicity, while in the second trimester the risk of fetal damage is minimal. Finally, if local anesthetics are to be administered in the third trimester, they should be administered in lower doses [32, 33].

The design of the present study only included dental students from a Peruvian university who were in their 3rd, 4th and 5th year of their professional career. The 1st and 2nd year students did not have the possibility of developing cognitive and procedural competencies in relation to pregnant patients since the curricula in these years only included general basic training courses. In addition, by carrying out the present study on students of different years from the same university, it was possible to control the curricular design variable and thus assess the progress of their knowledge as they develop the preclinical and clinical courses [34], since they were all trained with the same objective of articulating the characteristics, needs and perspectives of the professional practice with those of the training process under the same curricular design by competencies [35].

One of the limitations of the present study is that it was not possible to compare our results with those of previous studies, since these were very few [11, 17]. Another limitation was the fact that since the study was cross-sectional, it was not possible to assess whether the student’s knowledge improved over time. In addition, this research was limited to assessing the knowledge of students from a single university based in the capital city and one Peruvian province, so it is not possible to generalise the findings to the whole of Peru. However, this study is a starting point to identify the lack of knowledge in the prescription of drugs among dental students and, if necessary, to organise lectures and refresher and complementary courses to provide training on the proper use of drugs during pregnancy, with emphasis on the recommendation of these drugs for pregnant women according to the FDA classification to avoid possible maternal and fetal risks. Therefore, it would be advisable to replicate this study in other universities in Peru and other regions of the world. On the other hand, the validation of the instrument used was limited by the lack of a criterion analysis, as there was no gold standard test to assess the level of knowledge of pharmacological management of pregnant women in dental students. In addition, to reduce selection bias, potential confounding variables such as marital status, place of origin and area of residence were controlled for.

Further studies are also recommended to assess the knowledge of pharmacological management of pregnant women in students of different academic dental programmes at undergraduate and postgraduate level, while considering the associated variables in a logit model, to evaluate possible influential factors. Additionally, other confounding variables could be included, for example, socioeconomic level or training received in this subject in elective courses or whether the student has a direct family member who is a dentist or other variables in accordance with the social reality where the research is carried out.

## Conclusion

The level of knowledge about the pharmacological management of pregnant women among dental students at a Peruvian university was predominantly of fair level. In addition, it was observed that dental students under 24 years of age and those from the capital city were 44% less likely to have poor knowledge. It was also found that third- and fourth-year students were three times more likely to have poor knowledge than fifth year students. However, the variables sex, marital status and area of residence were not shown to be influential factors in the level of knowledge.

## Data Availability

All data analyzed during this study are available from the corresponding author on reasonable request (cesarcayorojas@gmail.com).

## Abbreviations

CI: Confidence interval
FDA: Food and Drug Administration
NSAID: Nonsteroidal anti-inflammatory drugs
OR: Odds ratio
STROBE: Strengthening the Reporting of OBservational studies in Epidemiology
SPSS: Statistical Package for the Social Sciences

## References

[1] Chacón P, Kanashiro C (2014). Salud bucal en el embarazo. Odontol Pediatr.

[2] Hartnett E, Haber J, Krainovich-Miller B, Bella A, Vasilyeva A, Lange-Kessler J (2016). Oral health in pregnancy. J Obstet Gynecol Neonatal Nurs.

[3] Li H-Y, Zhou Y-C, Zhou X-D (2018). Li-Wei Zheng; pharmacokinetics during pregnancy and safe medication of oral infectious diseases. West China J Stomatol.

[4] Alves RT, Ribeiro RA, Costa LR, Leles CR, Freire M, do CM y Paiva, SM. (2012). Oral care during pregnancy: attitudes of Brazilian public health professionals. Int J Environ Res Public Health.

[5] Lara Hernández A, Santiago Montealegre C (2016). Manejo Odontológico de mujeres embarazadas. Arch Inv Mat Inf.

[6] Ji Min L, Teo Jeon S (2017). Use of local anesthetics for dental treatment during pregnancy; safety for parturient. Dent Anesth Pain Med.

[7] Ajesh G, Ajwani S, Bhole S, Dahlen H, Reath J, Korda A (2017). Knowledge, attitude and practices of dentists towards oral health care during pregnancy: a cross sectional survey in New South Wales, Australia. Aust Dent J.

[8] Govindasamy R, Narayanan M, Balaji VR, Dhanasekaran M, Balakrishnan K, Christopher A (2018). Knowledge, awareness and practice between gynaecologistis, doctors and dentists in Madurai regarding the association between periodontitis and the results of pregnancy. J Indian Soc Periodontol.

[9] Sandoval Paredes J, Sandoval PC (2018). Uso de fármacos durante el embarazo. Horiz Med.

[10] Orueta Sánchez R, López Gil MJ (2011). Manejo de fármacos durante el embarazo. Inf Ter Sist Nac Salud.

[11] Guevara L, Falcón B (2018). Level of knowledge on the dental management of pregnanting patients, in students of stomatology of pre-clinic and clinic of the university alas Peruanas, Tacna subsidiary 2015. Revista Médica Basadrina.

[12] Corchuelo J, Mambuscay J. Survey of health undergraduates’ knowledge on pregnant women’ oral health and its association with pregnancy outcomes. Cali, 2015. Salud Uninorte. Barranquilla (Col.) 2018;34(3):652–66.

[13] Stanley AY, Durham CO, Sterrett JJ, Wallace JB (2019). Safety of over-the-counter medications in pregnancy. MCN Am J Matern Child Nurs.

[14] Acar S, Erol-Coskun H, Kaplan YC (2018). Use of antibiotics during pregnancy and the risk of major congenital malformations. Clin Pharmacol.

[15] Nicola W, Ouanounou A (2019). Pharmacotherapy for the pediatric dental patient. Comend Contin Educ Dent.

[16] Miklós R, Forgó K, Joób-Fancsaly Á, Ács N (2019). Várandósság és gyógyszerek. Fogorv Sz.

[17] Taybeh E, Kokash R, Talhouni A, Alsous M (2021). Knowledge about medicine use in pregnancy: a National Study among pharmacy students in Jordan. Curr Rev Clin Exp Pharmacol.

[18] Razban M, Giannopoulou C (2020). Knowledge and practices of Oral health care during pregnancy: a survey among Swiss dentists. Oral Health Prev Dent.

[19] Von Elm E, Altman DG, Egger M, Pocock SJ, Gøtzsche PC, Vandenbroucke JP (2008). The Strengthening the reporting of observational studies in epidemiology [STROBE] statement: guidelines for reporting observational studies. Gac Sanit.

[20] Kline RB (2005). Principles and practice of structural equation modeling.

[21] Cayo-Rojas CF, Soto-Castro L, Castro-Mena M, Medrano-Colmenares S, López-Gurreonero C, Córdova-Limaylla N (2022). Level of knowledge about metalloproteinases in dental students close to graduate from three universities in Peruvian capital city. Eur J Dent Educ.

[22] Togoo RA, Al-Almai B, Al-Hamdi F, Huaylah SH, Althobati M, Alqarni S (2019). Knowledge of pregnant women about pregnancy gingivitis and children Oral health. Eur J Dent.

[23] Kazma JM, van den Anker J, Allegaert K, Dallmann A, Ahmadzia HK (2020). Anatomical and physiological alterations of pregnancy. J Pharmacokinet Pharmacodyn.

[24] Instituto Nacional de Estadística e Informática. Comportamiento de los indicadores de mercado laboral a nivel nacional. Perú, 2021. Available from: https://www.inei.gob.pe/media/MenuRecursivo/boletines/03-informe-tecnico-empleo-nacional-abr-may-jun-2021.pdf.

[25] Icarte GA, Lávate HA (2016). Metodología para la revisión y Actualización de un diseño curricular de una carrera Universitaria. Formulario Univ.

[26] Alhemrani AE, Sreedharan J, Hassan S, Fanas A, Dsouza J, Reddy S (2022). Knowledge and perception about dental implants among undergraduate dental students and interns in UAE. J Int Dent Med Res.

[27] Donohue KE, Farber DL, Goel N, Parrino CR, Retener NF, Rizvi S, Dittmar PC (2021). Quality improvement amid a global pandemic: a virtual curriculum for medical students in the time of COVID-19. MedEdPORTAL.

[28] Tuha A, Gurbie Y, Hailu HG. Evaluation of knowledge and practice of pharmacy professionals regarding the risk of medication use during pregnancy in Dessie town, Northeast Ethiopia: a cross-sectional study. J Pregnancy. 2019:2186841. 10.1155/2019/2186841 [26]. 10.1155/2019/2186841 PMC6683770 31428474

[29] Carbonell C, Martos S, Rodríguez L, Hernández MJ, Jiménez C, Zurriaga Ó (2017). Consumo de medicamentos en el embarazo y riesgo de anomalías congénitas en la comunitat Valenciana. An Pediatr.

[30] Alfaro A, Unidad A, Alfaro AA, Navas IC, Magán Sanchez R, Jesús M (2018). Embarazo y salud oral. Rev Clín Med Fam.

[31] Wasylko L, Matsui D, Dykxhoorn SM, Rieder MJ, Weinberg S (1998). A review of common dental treatments during pregnancy: implications for patients and dental staff. J Can Dent Assoc.

[32] Ouanounou A, Haas DA (2016). Drug therapy during pregnancy: implications for dental practice. Br Dent J.

[33] Decloux D, Ouanounou A (2020). Local anaesthesia in dentistry: a review. Int Dent J.

[34] Cayo-Rojas C, Medrano-Colmenares S, Escurra-Estrada I, Ladera-Castañeda M, Agramonte-Rosell R, Cervantes-Ganoza L (2021). Epidemiological, preventive and healthcare knowledge about COVID-19, in dental students from three Peruvian universities. Edu Med Sup.

[35] Cayo-Rojas CF, Agramonte-Rosell RC (2020). Challenges of virtual education in dentistry in times of pandemic COVID-19. Rev Cuba Estomatol.

